# Determinants of mobile technology use and smartphone application interest in cancer patients

**DOI:** 10.1002/cam4.1660

**Published:** 2018-10-02

**Authors:** Nirupa Jaya Raghunathan, Deborah Korenstein, Qing S. Li, Emily S. Tonorezos, Jun J. Mao

**Affiliations:** ^1^ Department of Medicine Memorial Sloan Kettering Cancer Center New York New York; ^2^ Department of Medicine Weill Cornell Medical College New York New York

**Keywords:** cancer survivors, mobile technology, smartphone applications, supportive care, survivorship

## Abstract

**Background:**

Supportive care is a critical component of the treatment of cancer patients that is underutilized; patient lack of information about these services is an important barrier. Mobile technologies may be useful tools for delivering information, but cancer patient use of and interest in using them to learn about supportive care services have not been described. This study evaluates factors associated with cancer patient use of mobile technologies and interest in smartphone applications for information delivery about supportive care.

**Methods:**

We conducted a cross‐sectional survey among cancer patients from one urban academic hospital and 11 community hospitals. Patients self‐reported use of mobile technologies and interest in smartphone applications. Multivariate logistic analysis was used to identify determinants of mobile technology use and smartphone interest.

**Results:**

Among 631 participants, 466 (74%) reported regular use of mobile devices and 242 (39%) expressed an interest in supportive care information via smartphone applications. Patients under 45 were more likely to use a mobile device (Adjusted Odds Ratio [AOR] 6.8, 2.8‐16.9 95% CI,* P* < 0.001) and were interested in smartphone applications for delivery of information (AOR 3.2, 1.8‐5.9 95% CI,* P* < 0.001). Non‐white patients had similar use of mobile technology compared to whites but reported greater interest in smartphone application‐based information (AOR 3.4, 2.1‐5.5 95% CI,* P* < 0.001).

**Conclusion:**

Many patients expressed interest in smartphone application‐based information about supportive care services, especially those who are younger and non‐white. Future studies should investigate the characteristics of patients and smartphones applications that will optimize information delivery through a mobile technology platform.

## 1 INTRODUCTION

There are more than 15.5 million cancer survivors in the United States and this number is expected to rise to 20.3 million by 2026.^1^ Optimal care for this population goes beyond cancer treatment to include supportive care services to address common symptoms such as pain,^2^ fatigue,^3^ insomnia^4^, ^5^, and depressive symptoms.^6^ National Comprehensive Care Network (NCCN) guidelines for management of these symptoms recommend palliative care, cognitive behavioral therapy (CBT), mindfulness‐based stress reduction (MBSR), and supportive therapies. However, 30%‐60% of cancer patients have unmet supportive care needs^7^, ^8^ and these unmet needs may increase over time.^9^

Utilization of supportive care services among cancer survivors is low, ranging from 2% to 50%.^7^, ^10^ Barriers to use of these services often include lack of provider referral and lack of awareness.^10^ Cancer patients have been shown to have significant need for education and information around survivorship.^11^, ^12^ To better inform survivors of supportive care services, optimization of information delivery is needed.

Optimal methods for information delivery to cancer survivors are not clear. Patients in rural areas have expressed a preference for electronic formats for ongoing contact^13^ but there is little evidence informing optimal delivery mechanisms and there are organizational challenges with electronic communication of health care.^14^ Newer technologies, such as mobile phones, have been rapidly adopted with 77% of American adults owning a smartphone, up from 35% in 2011.^15^ In a general population, it has been shown that interest in mobile technologies was associated with greater depression and worse quality of life as well as greater self‐efficacy.^16^ Other conditions, such as pregnancy,^17^ smoking cessation^18^, and diabetes,^19^ have made use of mobile technologies to aid in information delivery in high‐need populations. Use of and interest in mobile technologies in the cancer survivor population specifically has not been described. These technologies are an opportunity for new and more efficient dissemination of supportive care information to cancer survivors.

Given the need for better information about symptom management among cancer survivors and the wide use of mobile technology, we set out to describe use and interest in smartphone applications for information delivery in the survivor population.

## 2 METHODS

### 2.1 Survey design and patients

We conducted a cross‐sectional survey study at the Abramson Cancer Center at the University of Pennsylvania in Philadelphia, PA, and Penn Cancer Network community hospitals in suburban and rural areas of Pennsylvania, New Jersey, and Delaware (Cape May Regional Hospital (Cape May, NJ); Chester Hospital (West Chester, PA); Community Medical Center (Toms River, NJ); Doylestown Hospital (Doylestown, PA); Kennedy Hospital, Kennedy Health Center (Cherry Hill, NJ); Kent General Hospital (Dover, DE); Lancaster General Health (Lancaster, PA); Milford Memorial Hospital (Milford, DE); Monmouth Medical Center (Longbranch, NJ); Pennsylvania Hospital (Philadelphia, PA); and Phoenixville Hospital (Phoenixville, PA) between December 2014 and September 2015. Research staff evaluated the eligibility criteria, approached patients during regular clinical visits, performed informed consent process and conducted the survey study. Patients were required to be 18 years of age or older, have a primary diagnosis of cancer, have a Karnofsky functional score of 60 or greater (ie ambulatory), understand written English, verbally indicate to the research staff that they felt physically well enough to complete a survey at the time of approach, and report experiencing nonzero pain (on a scale of 0‐10) in the last seven days. The Institutional Review Board of the University of Pennsylvania and the Scientific Review and Monitoring Committee of the Abramson Cancer Center approved the study protocol and surveys.

### 2.2 Outcomes

Mobile technologies were defined as smartphones, tablets, or text messaging. Patients reported frequency of use on a 5‐point Likert scale—never, less than once/month, less than once/week, at least once/week and daily. We defined regular use as patient reported utilization at least once per week or daily.

Interest in smartphone applications for information delivery format was measured on a 4‐point Likert scale, from very unimportant to very important; we defined a given communication method as “of interest” if patients rated it as important or very important.

Patients self‐reported date of cancer diagnosis and demographic factors including age, sex, race, education, and marital status. We dichotomized education to high school or less and college or above. We determined cancer type and stage from chart abstractions and dichotomized stage to metastatic and non‐metastatic. Patients reported if they had received surgery, chemotherapy, and/or radiation.

### 2.3 Statistical analysis

We performed statistical analysis using STATA software (Windows version 12.0, StatCorpLP, College Station, TX). We used univariate Chi^2^ testing to identify factors associated with mobile technology use and preference. We then conducted multivariate logistic regression analyses to identify factors associated with use and preference for mobile technology. We incorporated variables that were significant at *P* = 0.10 in the univariate Chi^2^ analysis. All analyses were two‐sided with p less than 0.05 indicating statistical significance in the multivariate model.

## 3 RESULTS

### 3.1 Demographics and clinical characteristics of study participants

Among the 631 participants, mean age was 60.3 years (range 23.1‐90.4), 415 (65.8%) were female, 521 (82.6%) white, 312 (53.8%) had non‐metastatic cancer, and approximately half (51.8%) were seen in community hospitals (Table 1). Most (n = 427, 68.2%) had completed at least some time in college, and 65.8% (n = 415) reported they were married or currently living with a partner. The most common cancer types were breast (n = 202, 32%), followed by thoracic, hematologic, and gastrointestinal. Nearly half (n = 303, 49.6%) of patients had been diagnosed in the 12 months prior to completing the survey, with 20.6% (n = 126) diagnosed within 12 to 36 months; 182 (29.8%) had been diagnosed more than 36 months before taking the survey. Most (n = 556, 88.1%) were treated with chemotherapy and about half had received surgery (n = 336, 53.3%) and radiation (n = 335, 53.1%).

**Table 1:** Characteristics of all survey participants (N = 631)

Characteristic	N	%
Age		
>65	225	35.7
56‐65	200	31.7
46‐55	136	21.6
≤45	70	11.1
Sex		
Female	415	65.8
Male	216	34.2
Race		
White	521	82.6
Non‐White	110	17.4
Education		
High school or less	199	31.8
College or above	427	68.2
Marital Status		
Not married	216	34.2
Married/living with partner	415	65.8
Cancer type		
Breast	202	32.0
Gastro‐Intestinal	81	12.8
Genito‐Urinary	36	5.7
Gynecologic	47	7.5
Head/Neck	53	8.4
Hematologic	93	14.7
Thoracic	93	14.7
Other	26	4.1
Cancer stage		
Non‐metastatic	312	53.8
Metastatic	268	46.2
Time since diagnosis		
≤12 mo	303	49.6
12‐36 mo	126	20.6
>36 mo	182	29.8
Surgery		
No	295	46.7
Yes	336	53.3
Chemotherapy		
No	75	11.9
Yes	556	88.1
Radiation		
No	296	46.9
Yes	335	53.1
Worst pain		
Mild	170	27.1
Moderate	148	23.6
Severe	309	49.3
Location of treatment		
Academic hospital	304	48.2
Community hospital	327	51.8

### 3.2 Use and determinants of use of mobile technologies

Among 631 respondents, 466 (73.9%) regularly used mobile technologies including smartphones (n = 356, 57%), tablets (n = 240, 38%), and text messaging (n = 418, 66%). Younger patients were more likely to report regular use of mobile technologies (91.4% for age ≤45 years, 89.0% for age 46‐55, 78.5% for age 56‐65 and 55.1% for age > 65) (Table 2). In addition, patients with at least some college education (79.6% vs 60.8% with high school education or less, *P* < 0.001), women (76.4% vs 69.0% of men, *P* = 0.045), and patients seen at an academic hospital (78.9% vs 69.1% of those treated at community hospitals, *P* = 0.005) were more likely to report regular use of mobile technologies. In multivariate analysis, patients under 45 years old were substantially more likely to use mobile technologies ([AOR] 6.8, 2.8‐16.9 95% CI, *P* < 0.001) than those aged older than 65 years (Table 3). Those with college or above education were also more likely to use mobile technologies than those with high school education or less (AOR 2.3, 1.5‐3.5 95% CI, *P* < 0.001). There was no difference in mobile technology use across races.

**Table 2:** Demographic/clinical factors and mobile device use and smartphone application interest

Characteristic	Mobile device usage			Smartphone interest		
	N	%	*P*‐value	N	%	*P*‐value
Age						
>65	124	55.1	**<0.001**	61	28.0	**<0.001**
56‐65	157	78.5		70	35.4	
46‐55	121	89.0		69	51.1	
≤45	64	91.4		42	60.0	
Sex						
Female	317	76.4	**0.045**	155	37.9	0.45
Male	149	69.0		87	41.0	
Race						
White	383	73.5	0.67	175	34.0	**<0.001**
Non‐White	83	75.5		67	63.2	
Education						
High school or less	121	60.8	**<0.001**	65	32.8	**0.036**
College or above	340	79.6		174	41.6	
Marital Status						
Not married	147	68.1	**0.017**	84	39.4	0.86
Married/living with partner	319	76.9		158	38.7	
Cancer type						
Breast	164	81.2	**0.010**	80	40.0	0.24
Gastro‐intestinal	65	80.2		37	45.7	
Genito‐urinary	28	77.8		17	47.2	
Gynecologic	34	72.3		18	39.1	
Head/Neck	39	73.6		15	29.4	
Hematologic	59	63.4		31	34.8	
Thoracic	59	63.4		30	32.6	
Other	18	69.2		14	53.8	
Cancer Stage						
Non‐metastatic	236	75.6	0.43	118	38.6	0.91
Metastatic	195	72.8		103	39.0	
Time since diagnosis						
<12 mo	216	71.3	0.16	114	37.9	0.88
12‐36 mo	101	80.2		49	39.8	
>36 mo	136	74.7		71	39.9	
Treatment—surgery						
No	195	66.1	**<0.001**	101	34.7	**0.041**
Yes	271	80.6		141	42.7	
Treatment—chemotherapy						
No	52	69.3	0.34	33	44.0	0.34
Yes	414	74.5		209	38.3	
Treatment—radiation						
No	217	73.3	0.77	116	40.1	0.58
Yes	249	74.3		126	37.9	
Worst pain						
Mild	128	75.3	0.26	57	34.8	0.18
Moderate	115	77.7		66	44.9	
Severe	219	70.9		117	38.2	
Location of treatment						
Academic hospital	240	78.9	**0.005**	128	43.0	0.051
Community hospital	226	69.1		114	35.3	

Values found to be significant to *P* < 0.05 are bolded.

**Table 3:** Multivariate analysis of mobile device use and smartphone interest

	Mobile device usage		Smartphone Interest	
	A.O.R. (95% C.I.)	*P*‐value	A.O.R. (95% C.I.)	*P*‐value
Age				
>65	1		1	
56‐65	2.8 (1.8‐4.4)	**<0.001**	1.2 (0.8‐1.9)	0.33
46‐55	6.2 (3.3‐11.5)	**<0.001**	2.7 (1.6‐4.3)	**<0.001**
≤45	6.8 (2.8‐16.9)	**<0.001**	3.2 (1.7‐5.9)	**<0.001**
Sex				
Female	1		1	
Male	0.7 (0.5‐1.1)	0.16	1.6 (1.1‐2.4)	**0.016**
Race				
White	1		1	
Non‐white	0.9 (0.5‐1.6)	0.72	3.4 (2.1‐5.5)	**<0.001**
Education				
High school or less	1		1	
College or above	2.3 (1.5‐3.5)	**<0.001**	1.4 (1.0‐2.1)	0.076
Marital Status				
Not married	1		1	
Married/living with partner	1.6 (1.0‐2.4)	**0.032**	1.0 (0.7‐1.5)	1
Treatment—surgery				
No	1		1	
Yes	1.6 (1.1‐2.4)	**0.022**	1.5 (1.1‐2.2)	**0.021**
Location of treatment				
Academic hospital	1		1	
Community hospital	0.8 (0.6‐1.3)	0.43	1.0 (0.7‐1.4)	0.99

Values found to be significant to *P* < 0.05 are bolded.

### 3.3 Determinants of interest in smartphone applications

Overall, 242 (39%) patients expressed interest in smartphone applications to learn about supportive care services, with more younger patients reporting this interest (60.0% for age ≤45 vs 28.0% for age >65, *P* < 0.001). There was no significant difference in interest between genders, with 37.9% of women and 41.0% of men expressing interest in information delivery via smartphone application (*P* = 0.45). Interest varied by race with 34.0% of white patients and 63.2% of non‐white patients expressing interest in information delivery of supportive care services by smartphone application (*P* < 0.001). Of those with a high school education or less, 32.8% expressed an interest in smartphone application‐delivered information compared with while 41.6% of those with college or above (*P* = 0.036; Table 2).

In multivariate logistic analysis, younger age (AOR 3.2 for age ≤45 compared to >65, 1.7‐5.9 95% CI, *P* < 0.001), non‐white race (AOR 3.4, 2.1‐5.5 95% CI, *P* < 0.001), and male gender (AOR 1.6, 1.1‐2.4 95% CI, *P* = 0.016) were associated with an interest in receiving supportive care information through smartphone applications. Interest in information via smartphone app was similar across education groups (Table 3).

## 4 DISCUSSION

Our data demonstrate high levels of mobile technology use, particularly among younger cancer survivors. Non‐white patients, younger patients and male patients reported more interest in delivery of information through smartphone applications. While overall mobile technology use is similar to that reported for the general population,^15^ variations in interest and reported use among patients have implications for the design of mobile technology approaches to information delivery regarding supportive care.

Adolescent and young adult cancer patients have been found to have poorer physical and emotional well‐being compared to healthy controls.^20^ Greater awareness of supportive care services could ameliorate this outcome. It is not surprising to find younger patients have an interest in smartphone applications for the delivery of important information regarding supportive care services. In the adolescent and young adult (AYA) population, the proportion reporting smartphone use approaches 94%.^15^ Many may not remember a time before the internet and smartphones.^21^ Though the AYA survivor often uses mobile and internet technology to guide healthy behaviors, Mooney et al^22^ showed that much of the information they found did not meet their needs. Our results encourage further evaluation of mobile applications to educate this less‐informed population about potential supportive care interventions.

Our findings with regards to race are interesting and are reflective of other studies. In a national telephone survey study of cancer information seeking behavior, social determinants of race, ethnicity and social class affected preference for information sources.^23^ It is known that non‐white cancer survivors experience lower health‐related quality of life (HRQOL),^24^ are more likely to be obese^25^ and experience a physical limitation^26^ and poorer patient‐provider communication.^27^ More specific communications tailored to characteristics such gender, language, health literacy, and culture may improve uptake of recommended interventions.^28^, ^29^, ^30^ Our study shows a significant interest in smartphone applications, particularly in a non‐white population. Increased use of technology, such as online patient portals, for communication^31^, ^32^ indicates potential to utilize mobile technology to increase awareness of supportive care services, decrease barriers, and improve health outcomes in cancer patients.

There are several limitations to our study. In this survey‐based study, there is the potential for recall bias and results should be interpreted with caution. However, survey responses regarding preference require little recall and are relevant for guiding information delivery. Selection bias is possible although our recruitment across academic and community centers and high response rate are reassuring. The survey tool was developed and administered in an English‐speaking population, which may lead to underrepresentation of certain cultural groups and results may not be generalizable to a non‐English speaking population. Nonetheless, our study indicates there are differences among a general cancer population in mobile technology use and smartphone application interest.

Our results point to an interest in information delivery via smartphone applications, particularly in younger and non‐white populations. There is still a gap in understanding why there is an interest in mobile technology for information delivery and how to further improve smartphone applications to serve in supportive care. With further research, our findings suggest it is possible to optimize mobile technology to aid in delivery of evidence‐based recommendations to underserved populations.

## CONFLICTS OF INTEREST

There are no conflicts of interest disclosures from any authors.
